# A speed optimization model for connected and autonomous vehicles at expressway tunnel entrance under mixed traffic environment

**DOI:** 10.1371/journal.pone.0314044

**Published:** 2024-12-09

**Authors:** Jianrong Cai, Yang Liu, Zhixue Li

**Affiliations:** 1 School of Civil Engineering, Hunan City University, Yiyang, China; 2 School of Traffic and Transportation, Lanzhou Jiaotong University, Lanzhou, China; 3 Hunan City University Design and Research Institute Co., Ltd, Changsha, China; Southwest Jiaotong University, CHINA

## Abstract

Rear-end collisions frequently occurred in the entrance zone of expressway tunnel, necessitating enhanced traffic safety through speed guidance. However, existing speed optimization models mainly focus on urban signal-controlled intersections or expressway weaving zones, neglecting research on speed optimization in expressway tunnel entrances. This paper addresses this gap by proposing a framework for a speed guidance model in the entrance zone of expressway tunnels under a mixed traffic environment, comprising both Connected and Autonomous Vehicles (CAVs) and Human-driven Vehicles (HVs). Firstly, a CAV speed optimization model is established based on a shooting heuristic algorithm. The model targets the minimization of the weighted sum of the speed difference between adjacent vehicles and the time taken to reach the tunnel entrance. The model’s constraints incorporate safe following distances, speed, and acceleration limits. For HVs, speed trajectories are determined using the Intelligent Driver Model (IDM). The CAV speed optimization model, represented as a mixed-integer nonlinear optimization problem, is solved using A Mathematical Programming Language (AMPL) and the BONMIN solver. Safety performance is evaluated using Time-to-Collision (TTC) and speed standard deviation (SD) metrics. Case study results show a significant decrease in SD as the CAV penetration rate increases, with a 58.38% reduction from 0% to 100%. The impact on SD and mean TTC is most pronounced when the CAV penetration rate is between 0% and 40%, compared to rates above 40%. The minimum TTC values at different CAV penetration rates consistently exceed the safety threshold TTC*, confirming the effectiveness of the proposed control method in enhanced safety. Sensitivity analysis further supports these findings.

## 1 Introduction

Road traffic safety remains a critical global issue. According to data from the World Health Organization (WHO), approximately 1.3 million lives are lost every year due to road traffic crashes [1]. Research indicates that the average annual accident lethality rate on expressways is 1.33 times higher than on highways [2]. Notably, expressway tunnels are particularly accident-prone, with a higher proportion of fatalities and severe injuries [3, 4]. Statistics show that accident rates in the entrance zones of expressway tunnels are approximately five times higher than in other zones [5]. Furthermore, data from the Norway Highway Bureau reveals that 63.7% of accidents occur in the tunnel entrance zones [6].

During daylight hours, the sharp decrease in illumination upon entering a tunnel, commonly known as the visual "black-hole effect," is a major safety concern. The sudden change in brightness requires vehicles to decelerate before entering the tunnel, which significantly increases the likelihood of rear-end collisions [7, 8]. Research has shown that this visual effect poses a serious risk to safety in the transition zones at tunnel entrances [9]. Moreover, after an accident occurs, delayed warnings and insufficient deceleration time for trailing vehicles can contribute to secondary accidents. These secondary accidents often result in more severe injuries and greater property damage than standard traffic incidents [10]. Consequently, providing speed guidance for vehicles approaching tunnel entrance zones is crucial for ensuring safe driving conditions.

In recent years, Vehicle-to-Everything (V2X) communication and sensing technologies have emerged as revolutionary technologies. By facilitating two-way communication between vehicle and vehicle/infrastructure, these technologies enable the deployment of speed guidance or speed optimization schemes [11]. Optimizing vehicle speed can lead to substantial improvements in traffic flow, fuel efficiency, environmental impact, and overall safety [12–14]. Research on speed optimization falls into two main categories based on different traffic scenarios: intermittent traffic flow (e.g., at signalized intersections) and continuous traffic flow (e.g., on urban highways or expressways).

In the research on intermittent traffic flow speed optimization, the primary focus is on optimizing vehicle speeds upstream of intersections through signal timing schemes to achieve energy conservation, emissions reduction, and enhance traffic efficiency [15, 16]. Numerous studies have focused on the research of guiding vehicle speeds at signalized intersections [17]. Some studies have relied on fixed signal timing plans for vehicle speed optimization [18]. For example, He et al. (2015) proposed a multi-stage optimal control model for the optimization of vehicle speed trajectories at signalized intersections [19]. In addition, some studies have optimized signal control and CAVs’ speed trajectories by adopting signal-trajectory joint control strategies [20, 21]. For instance, Yu et al. (2018) present a model that optimizes CAV speed trajectories and urban intersection traffic signal (i.e., phase sequences, the initiation of green lights, and the duration of each phase) within a unified framework to minimize delays [22]. Furthermore, beyond individual signalized intersections, some studies have addressed the optimization of vehicle speeds at successive signalized intersections [23, 24]. For example, Lu et al. (2019) introduced a method for optimizing speed at successive signalized intersections to reduce vehicle fuel consumption and emissions [25].

In the foreseeable future, a mixed traffic environment with the coexistence of CAV and HV will persist. Several researchers have explored the optimization of vehicle trajectories at intersections within mixed traffic environment [26, 27]. For example, Zhao et al. proposed an innovative approach to model vehicle behavior and optimize trajectories, specifically highlighting the two-dimensional movement of vehicles at intersections [28, 29]. Tajalli et al. (2022) proposed a methodology for optimizing vehicle trajectories and signal timing at intersections with mixed traffic flow [30]. Yao et al. (2024) proposed a comprehensive vehicle-following-based method for full-sample trajectory reconstruction, which effectively reconstructs vehicle trajectories across varying traffic densities [31].

Research on continuous traffic flow optimization focuses on guiding vehicle speeds to minimize speed fluctuations and reduce conflicts during merging [32]. Hu et al. (2019) proposed a control algorithm for freeway merging zones that simultaneously optimizes lane-changing and car-following trajectories within a CAV environment [33]. Sun et al. (2020) developed a cooperative decision-making mechanism to facilitate efficient and smooth ramp merging in mixed traffic [34]. Ko et al. (2020) propose speed harmonisation and merge control strategies to alleviate highway traffic congestion [35].

In addition, some studies have examined the impact of vehicle speed on energy conservation and emission reduction on highways [34]. For example. Bray et al. (2022) investigated the fuel consumption of autonomous trucks at various target speeds on highways [36]. Rui et al. (2023) introduced a truck platooning optimal speed control model on highways, focusing on minimizing overall travel time and reducing fuel consumption within truck platoons [37].

In summary, existing research on speed guidance primarily focuses on urban intersections. However, applying speed optimization models developed for intersections to highway tunnel scenarios presents significant challenges for two main reasons. First, at intersections, signal timing provides a clear basis for adjusting vehicle speed. Speed guidance models, for example, offer differentiated guidance based on whether vehicles encounter red or green signals when approaching the intersection [38]. In contrast, at expressway tunnel entrances, the absence of traffic signals removes a similar basis for speed adjustment. Second, vehicle speeds on urban roads are generally slower than on expressways, allowing for quicker deceleration and requiring shorter time and space to slow down. Contrastingly, rapid deceleration can lead to severe accidents, and the minimum speed requirements and prohibition of stopping add further complexity. These factors create significant differences in the modeling and solving of speed optimization between urban signalized intersections and expressway speed guidance.

Research on speed guidance for continuous traffic flow has largely concentrated on weaving zones of expressways, but there is a noticeable lack of studies addressing speed guidance in expressway tunnel entrance zones. Moreover, some studies under the assumption of 100% CAV penetration. For instance, Li et al. (2018) developed a simplified traffic smoothing model for a 100% CAV scenario to guide vehicle movements on a highway segment [32]. However, achieving full CAV penetration is unlikely soon. Consequently, technologies developed exclusively for such environments may not be feasible for widespread implementation in the short term.

Existing speed optimization models mainly focus on urban signal-controlled intersections or expressway weaving zones, often overlooking speed optimization at expressway tunnel entrances. To address these gaps, this paper contributes to the literatures as follows:

Firstly, we propose a novel CAVs speed optimization model. The model is designed with constraints that account for the "black hole effect," where vehicles must decelerate before entering an expressway tunnel while still adhering to minimum speed limits. Unlike previous studies, which focus mainly on efficiency, our model prioritizes both safety and efficiency. The objective function minimizes the weighted sum of speed differentials between adjacent vehicles and the time required to reach the tunnel entrance. A sensitivity analysis is also conducted to explore the impact of varying weight values.

Secondly, this study utilizes the Intelligent Driver Model (IDM) to calculate the speed trajectories of HVs. However, since the IDM model does not inherently satisfy expressway minimum speed limits, additional constraints are introduced to improve its performance. Furthermore, we propose an integrated model framework that combines the CAV speed optimization model with the improved IDM model in a mixed traffic environment. This unified framework records the optimization results of the front vehicle, including speed and displacement per second, and uses these as input conditions for the rear vehicle’s speed optimization. Safety metrics, such as Time to Collision (TTC) and speed standard deviation (SD), are employed to assess the safety performance of the speed optimization model under varying CAV penetration rates.

The remainder of this study is organized as follows: Section 2 defines the problem statement. Section 3 introduces the development of the speed optimization model for CAVs and the IDM for HVs. Section 4 discusses the model solution process. Section 5 analyzes the impact of CAVs on traffic safety through a case study. Finally, Section 6 presents conclusions and suggestions for future research.

## 2 Problem statement

**Fig 1:**
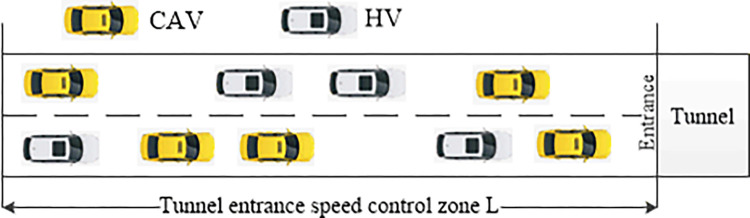
The mixed traffic flow of CAV and HV in tunnel entrance zone.

In the tunnel entrance zone, vehicles are required to decelerate before entry, which increases the risk of rear-end collisions. Fig 1 illustrates the mixed traffic flow of CAV and HV in the expressway tunnel zone. The driving speed of HVs in this zone can be calculated using the IDM for car-following scenarios. The primary focus of this paper is to address the first key issue: how to develop a CAV speed trajectory optimization model that enhances the safety of mixed traffic flow. This involves optimizing the speed of CAVs while taking into account the necessity for HVs to decelerate before entering the tunnel. It is important to note that the front vehicle for a CAV could be either an HV or another CAV. Additionally, this paper addresses the second issue of evaluating safety impacts at varying CAV penetration rates within the traffic flow.

**Fig 2:**
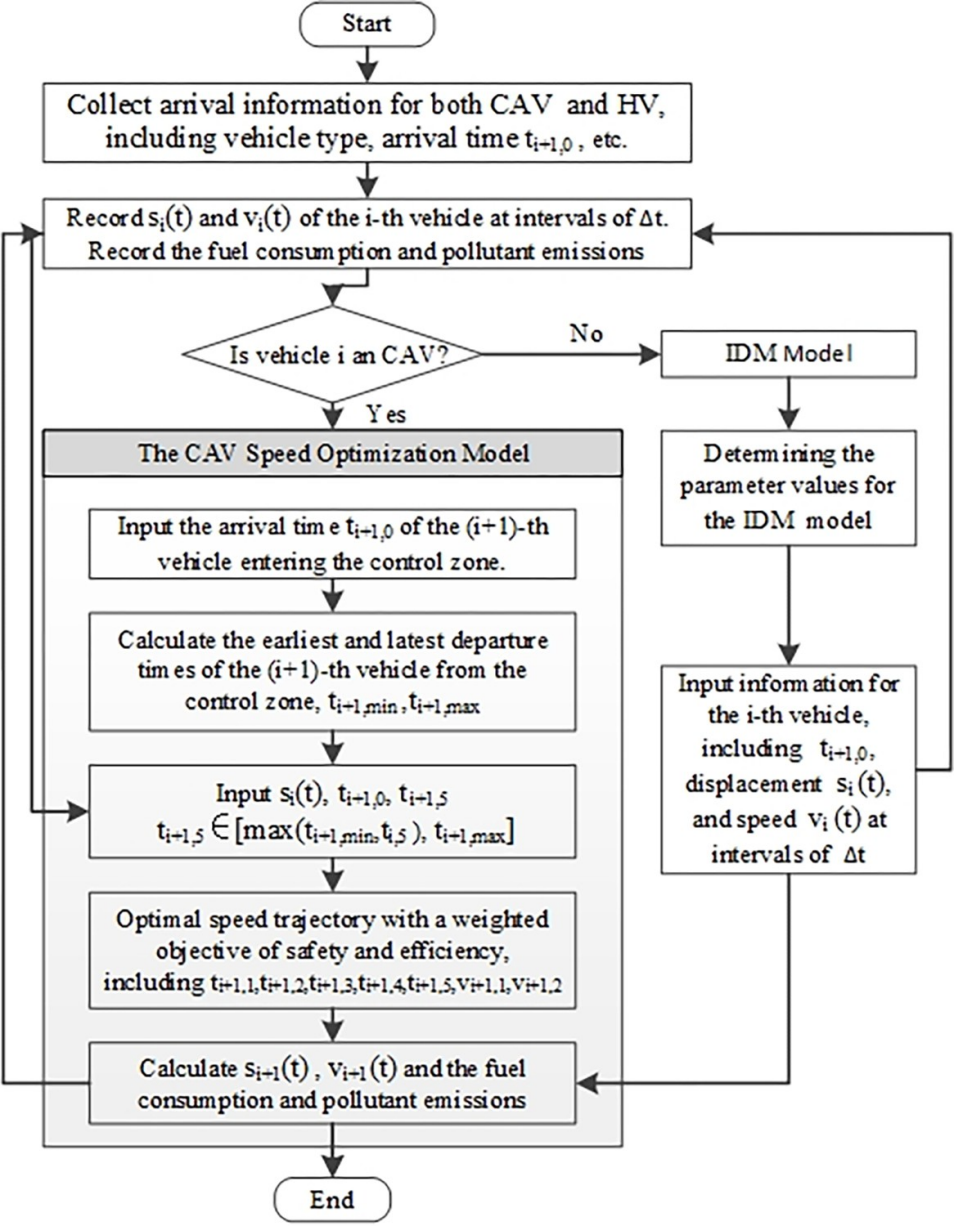
Model framework.

Fig 2 is the model framework. Before vehicles enter the tunnel control zone, their arrival information—including position, speed, and vehicle type (CAV or HV)—is collected. For HVs, the IDM is applied to calculate their driving speed based on the speed trajectory of the preceding vehicle in the same lane, with the results recorded accordingly. For CAVs, the established CAV speed optimization model is used to optimize their speed trajectory. Initially, the time range for the vehicle to reach the tunnel entrance is computed based on the recorded data, including the vehicle’s arrival time within the control zone and its maximum acceleration. The current CAV’s speed is then optimized under safety constraints, and the optimized speed is recorded as input for the following vehicle.

## 3 Methodology

### 3.1 Parameter description

The descriptions of the notations is summarized in Table **1**:

**Table 1:** Notation description.

Set	
*I*_*l*_	A collection of all vehicles in lane *l*
*I*_*l*,*CAV*_	A collection of CAVs on lane *l*
*I*_*l*,*HV*_	A collection of HVs on lane *l*
Parameter	
*ω*	the weight coefficient
*t*_*i*,0_	The time when vehicle *i* enters the speed control zone
*V*_*m*_,*v*_*m*_	The upper and lower limits of speed, respectively
*v*_*d*_	The vehicle’s desired speed
*δ*	The acceleration exponent
*a*_*d*_	The comfortable deceleration
*T*	The safe time headway
*s*_0_	The minimum distance headway
*t*_*i*,*M*_,*t*_*i*,*m*_	The maximum and the minimum travel time, respectively
Δ*t*	The time interval
Variable	
*v*_*i*_(*t*)	The speed of the vehicle *i* at time *t*
Δ*φ*_*i*,*i*−1_(*t*)	The absolute value of the speed difference between adjacent vehicles
Δ*v*_*i*,*i*−1_(*t*)	The speed difference between adjacent vehicles
*s*_*i*_(*t*)	The displacement of vehicle *i* at time *t*
Δ*s*_*i*,*i*−1_(*t*)	The distance gap between adjacent vehicles
*a*_*i*,1_ *a*_*i*,2_	The deceleration of vehicle *i* in the first and second stages, respectively
*t*_*i*,*k*_	The time when vehicle *i* begins or ends different velocity stages *k*,*k*∈{1,2,…,5}

### 3.2 The CAV speed optimization model

**Objective function:** The CAV speed optimization model aims for a weighted combination of safety and efficiency, as illustrated in Eq (1):

$$\min(\omega ∙\sum_{t=t_{i,0}}^{t_{i,5}}{\Delta \varphi }_{i,i-1}(t)+(1-\omega )∙t_{i,5}),\;\forall i\in I_{l,CAV},i-1\in I_{l}$$ (1)

where *ω* is the coefficient between safety and efficiency, with 0≤*ω*≤1; Δ*φ*_*i*,*i*−1_(*t*) is the absolute value of the speed difference between vehicle *i* and vehicle *i*−1, satisfying the Eqs (2)–(3):

$${\Delta \varphi }_{i,i-1}(t)\geq v_{i-1}(t)-v_{i}(t),\forall t\in \{t_{i,0},\ldots t_{i,5}\},i\in I_{l,CAV},i-1\in I_{l}$$ (2)

$${\Delta \varphi }_{i,i-1}(t)\geq -(v_{i-1}(t)-v_{i}(t)),\forall t\in \{t_{i,0},\ldots t_{i,5}\},i\in I_{l,CAV},i-1\in I_{l}$$ (3)

**Fig 3:**
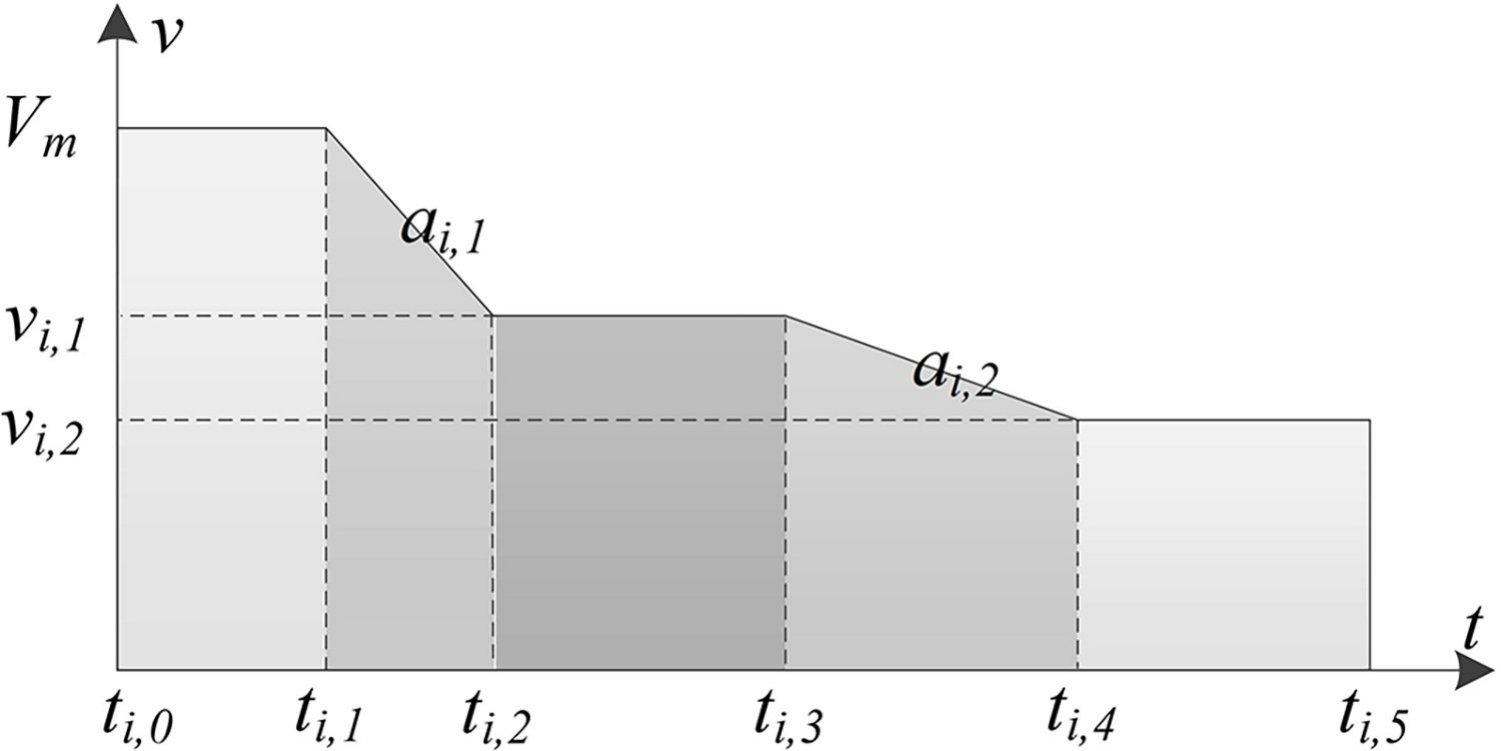
Five-stage speed adjustment.

**Speed and Acceleration Constraints:** In this study, a CAV speed optimization model is developed using the shooting heuristic algorithm [39, 40]. As shown in Fig 3, the model restricts each trajectory to a maximum of five distinct stages. After maintaining a constant speed for a certain period, the vehicle decelerates at a rate of *a*_*i*,1_. It then maintains another constant speed for a period before decelerating again at a rate of *a*_*i*,2_.

While this modelling approach reduces the feasible region for speed, resulting in a slight compromise in solution optimality, we find it reasonable for two key reasons:

Firstly, in practice, vehicles entering an expressway tunnel do not frequently accelerate or decelerate. Thus, assuming a constant deceleration rate during each phase introduces minimal errors in shaping the optimal trajectory. Secondly, since neighboring CAVs follow each other, their optimal trajectories are expected to exhibit similar deceleration profiles. When a CAV follows an HV, the optimal CAV deceleration is determined based on the HV’s speed trajectory. Secondly, since neighboring CAVs follow each other, their optimal trajectories are expected to exhibit similar deceleration profiles. When a CAV follows an HV, the optimal CAV deceleration is determined based on the HV’s speed trajectory.

The CAV speed optimization model primarily optimizes the initiation time of deceleration, the deceleration value, the duration of deceleration, and the time of arrival at the tunnel entrance. The constraints include specifying that vehicle *i* decelerates during the time intervals from *t*_*i*,1_ to *t*_*i*,2_ and from *t*_*i*,3_ to *t*_*i*,4_ at decelerations *a*_*i*,1_ and *a*_*i*,2_, respectively, as depicted in Eqs (4)–(5). The ranges for velocity and deceleration are defined by Eqs (6)–(7).


$$v_{i,1}=v_{M}-(t_{i,2}-t_{i,1})∙a_{i,1},\;\forall i\in I_{l,CAV}$$ (4)

$$v_{i,2}=v_{i,1}-(t_{i,4}-t_{i,3})∙a_{i,2},\;\forall i\in I_{l,CAV}$$ (5)

$$0\leq a_{i,1},a_{i,2}\leq a_{M},\;\forall i\in I_{l,CAV}$$ (6)

$$v_{m}\leq v_{i,2}\leq v_{i,1}\leq V_{m},\;\forall i\in I_{l,CAV}$$ (7)

**Time constraints: ***t*_*i*,1_,*t*_*i*,2_,*t*_*i*,3_,*t*_*i*,4_ represent the time when vehicle *i* begins or ends at different velocity stages, respectively. Here, *t*_*i*,1_ and *t*_*i*,2_ denote the time of start and completion of deceleration with *a*_*i*,1_ *t*_*i*,3_ and *t*_*i*,4_ denote the time of start and completion of deceleration with *a*_*i*,2_ *a*_*i*,1_,*a*_*i*,2_,*a*_*M*_ represent the value of deceleration, all without negative signs. The temporal relationships between different time are described by Eq (8):

$$t_{i,0}\leq t_{i,1}\leq t_{i,2}\leq t_{i,3}\leq t_{i,4}\leq t_{i,5},\;\forall i\in I_{l,CAV}$$ (8)

On the expressway, the vehicle traveling speed cannot fall below the minimum speed. Therefore, there exist the maximum travel time *t*_*i*,*M*_ and the minimum travel time *t*_*i*,*m*_ for the vehicle to cover a distance of *L*. These are calculated by Eqs (9)–(10):

$$t_{i,M}=t_{i,0}+(v_{M}-v_{m})/a_{M}+(L-({v_{M}}^{2}-{v_{m}}^{2})/(2∙a_{M}))/v_{m},\;\forall i\in I_{l,CAV}$$ (9)

$$t_{i,m}=t_{i,0}+(v_{M}-v_{m})/a_{M}+(L-({v_{M}}^{2}-{v_{m}}^{2})/(2∙a_{M}))/v_{M},\;\forall i\in I_{l,CAV}$$ (10)

where *L* is the length of the tunnel entrance speed control zone;

The arrival time of vehicle *i* at the tunnel entrance is jointly determined by the time the front vehicle takes to reach the tunnel entrance *t*_*i*,1,5_,*t*_*i*,*M*_ and *t*_*i*,*m*_, as constrained by Eq (11):

$$\max\left(t_{i,m},t_{i-1,5}+T\right)\leq t_{i,5}\leq t_{i,M},\;\forall i\in I_{l,CAV},i-1\in I_{l}$$ (11)

where *T* represents the safety headway time.

The speed *v*_*i*_(*t*), displacement *s*_*i*_(*t*), and acceleration *a*_*i*_(*t*) of the CAV at time *t* are calculated by Eqs (12)–(14):

$$v_{i}(t)=\left\{ \begin{array}{c} v_{M},\;t_{i,0}\leq t<t_{i,1}\\ v_{M}-(t-t_{i,1})∙a_{i,1},t_{i,1}\leq t<t_{i,2}\\ v_{i,1},\;t_{i,2}\leq t<t_{i,3}\\ v_{i,1}-(t-t_{i,3})∙a_{i,2},t_{i,3}\leq t<t_{i,4}\\ v_{i,2},\;t_{i,4}\leq t\leq t_{i,5} \end{array},\;\forall i\in I_{l,CAV}\right.$$ (12)

$$a_{i}(t)=\left\{ \begin{array}{c} 0,\;t_{i,0}\leq t<t_{i,1}\\ a_{i,1},\;t_{i,1}\leq t<t_{i,2}\\ 0,\;t_{i,2}\leq t<t_{i,3}\\ a_{i,2},\;t_{i,3}\leq t<t_{i,4}\\ 0,\;t_{i,4}\leq t\leq t_{i,5} \end{array},\;\forall i\in I_{l,CAV}\right.$$ (13)

$$s_{i}(t)=\left\{ \begin{array}{c} v_{M}∙(t-t_{i,0}),\;t_{i,0}\leq t<t_{i,1}\\ {s_{i}(t}_{i,1})+v_{M}∙(t-t_{i,1})-1/2∙a_{i,1}∙{\left(t-t_{i,1}\right)}^{2},t_{i,1}\leq t<t_{i,2}\\ {s_{i}(t}_{i,2})+v_{i,1}∙(t-t_{i,2}),t_{i,2}\leq t<t_{i,3}\\ {s_{i}(t}_{i,3})+v_{i,1}∙(t-t_{i,3})-1/2∙a_{i,2}∙{\left(t-t_{i,3}\right)}^{2},t_{i,3}\leq t<t_{i,4}\\ {s_{i}(t}_{i,4})+v_{i,2}∙(t-t_{i,4}),\;t_{i,4}\leq t\leq t_{i,5} \end{array},\forall i\in I_{l,CAV}\right.$$ (14)

The displacement of the vehicle when it reaches the tunnel entrance is equal to the length of the control zone, as constrained by Eq (15):

$$L={s_{i}(t}_{i,5}),\forall i\in I_{l,CAV}$$ (15)

**Safety Constraint:** When calculating the velocity trajectory of vehicle *i*, the speed and displacement of the front vehicle *i* are considered as known inputs. Overtaking is not allowed within the control zone, meaning that at any given moment, the displacement of the front vehicle must be greater than that of the following vehicle, as constrained by Eq (16):

$$s_{i-1}(t)\geq s_{i}(t)+s_{0},t\in \{t_{i,0},\ldots ,t_{i,5}\},\forall i\in I_{l}$$ (16)

Where *s*_0_ is the minimum headway distance.

### 3.3 The intelligent driver model

This paper addresses mixed traffic consisting of both CAVs and HVs. The speed trajectory of CAVs is computed using the speed optimization model outlined in Section 3.2, while the speed trajectory of HVs is determined using the Intelligent Driver Model (IDM) [41]. The IDM has relatively few parameters and variables, each with clear physical significance [41]. In line with the majority of studies that utilize the IDM for analyzing human-driven vehicle trajectories, our study similarly did not account for the heterogeneous driving behaviors exhibited by individual human drivers [42, 43]. The acceleration *a*_*i*_(*t*) of an HV at time *t* is computed using Eq (17), where s*(*v*_*i*_(*t*),Δ*v*_*i*,*i*−1_(*t*)) in Eq (17) represents the desired following distance, calculated by Eq (18).


$$a_{i}(t)=a_{M}∙[1-{\left(\frac{v_{i}(t)}{v_{d}}\right)}^{\delta }-{\left(\frac{s^{*}(v_{i}(t),{\Delta v}_{i,i-1}(t))}{s_{i,i-1}(t)}\right)}^{2}],\;\forall i\in I_{l,HV},i-1\in I_{l}$$ (17)

$$s^{*}(v_{i}(t),{\Delta v}_{i,i-1}(t))=max(0,v_{i}(t)∙T+\frac{v_{i}(t)∙(v_{i}(t)-v_{i-1}(t))}{2\sqrt{a_{M}∙a_{d}}}),\;\forall i\in I_{l,HV},i-1\in I_{l}$$ (18)

Where, *a*_*M*_ denotes maximum acceleration of the vehicle; *v*_*d*_ is vehicle desired speed; *δ* is acceleration exponent. The acceleration exponent *δ* characterizes how acceleration decreases. A larger *δ* implies a more aggressive acceleration and deceleration process for the vehicle. *s*_*i*,*i*−1_(*t*) represents distance gap between adjacent vehicles; s*(*v*_*i*_(*t*),Δ*v*_*i*,*i*−1_(*t*)) denotes the expected following distance. *a*_*d*_ is comfortable deceleration of the vehicle; *T* is safe time headway; *s*_0_ is minimum distance headway.

The speed calculated by the IDM for HVs does not meet the expressway’s minimum speed limit. Therefore, we have improved the IDM model by introducing a constraint. This constraint assesses whether the vehicle’s speed per second exceeds the minimum speed. If the speed after deceleration is below the minimum, the vehicle travels at the minimum speed without further deceleration. The constraint is shown in Eq (19):

$$if\;v_{i}(t)\geq v_{m}\;then\;v_{i}(t)=v_{i}(t-1)-a_{i}(t)∙\Delta t\;else\;v_{i}(t)=v_{m}\forall i\in I_{l,HV}$$ (19)

Where Δ*t* is the time interval.

## 4 Model solution

The CAV speed optimization model is a Mixed Integer non-linear programming model (MINLP). Eqs (12)–(14) represent piecewise functions, and by utilizing 0–1 variables, these piecewise linear functions can be expressed in a linear form. Suppose that a piecewise linear function *f*(*x*) has break points *b*_1_,*b*_2_,…*b*_*n*_. For some *k* (*k* = 1,2,…,n−1), *b*_*k*_≤x≤*b*_*k*+1_. Then, for some number *z*_*k*_ 0≤*z*_*k*_≤1,*x* maybe written as

$$x=z_{k}∙b_{k}+(1-z_{k})∙b_{k+1}$$ (20)

Because *f*(*x*) is linear for *b*_*k*_≤x≤*b*_*k*+1_, we may write

$$f(x)=z_{k}∙f(b_{k})+(1-z_{k})∙f(b_{k+1})$$ (21)

Referring to the method of linearizing piecewise functions, let’s take Eq 12 as an example of linearization. The linearized formula is as follows:

$$y_{i,k}(t)=0\;or\;1,\forall k\in \{1,2,3,4,5\}$$ (22)

$$0\leq y_{i,k}(t)\leq 1,\forall k\in \{1,2,3,4,5\}$$ (23)

$$z_{i,1}(t)\leq y_{i,1}(t)$$ (24)

$$z_{i,2}(t)\leq y_{i,1}(t)+y_{i,2}(t)$$ (25)

$$z_{i,3}(t)\leq y_{i,2}(t)+y_{i,3}(t)$$ (26)

$$z_{i,4}(t)\leq y_{i,3}(t)+y_{i,4}(t)$$ (27)

$$z_{i,5}(t)\leq y_{i,4}(t)+y_{i,5}(t)$$ (28)

$$z_{i,6}(t)\leq y_{i,5}(t)$$ (29)

$$z_{i,1}(t)+z_{i,2}(t)+z_{i,3}(t)+z_{i,4}(t)+z_{i,5}(t)+z_{i,6}(t)=1$$ (30)

$$y_{i,1}(t)+y_{i,2}(t)+y_{i,3}(t)+y_{i,4}(t)+y_{i,5}(t)=1$$ (31)

$$t=z_{i,1}(t)∙t_{i,0}+z_{i,2}(t)∙t_{i,1}+z_{i,3}(t)∙t_{i,2}+z_{i,4}(t)∙t_{i,3}+z_{i,5}(t)∙t_{i,4}+z_{i,6}(t)∙t_{i,5}$$ (32)

$$v_{i}(t)=z_{i,1}(t)∙v_{M}+z_{i,2}(t)∙v_{M}+z_{i,3}(t)∙v_{i,1}+z_{i,4}(t)∙v_{i,1}+z_{i,5}(t)∙v_{i,2}+z_{i,6}(t)∙v_{i,2}$$ (33)

Despite linearizing some nonlinear constraints, such as piecewise functions, certain nonlinear constraints remain challenging to linearize, particularly the multiplication of two variables in Eqs (4)–(5). As a result, the CAV speed optimization model remains a Mixed-Integer Nonlinear Programming (MINLP) problem. To solve this model, we use Python to interface with A Mathematical Programming Language (AMPL) and the Bonmin (Basic Open-source Nonlinear Mixed Integer Programming) solver. AMPL is a powerful tool for formulating and solving optimization problems, while Bonmin is an open-source solver specifically designed to address general MINLP problems. The optimization problem was solved on a computer with a Win-10 64-bit operating system, Intel(R) Core (TM) i9-10900K CPU, 3.70 GHz, and 64 GB random access memory (RAM).

## 5 Case study

### 5.1 Results and discussions

Collisions in expressway tunnel zones primarily occur within the same lane. As a result, this paper focuses on a single-lane scenario in the case analysis when evaluating the safety of expressway tunnel entrance zones, excluding lane-changing behavior. The control zone is a 500 meter road segment leading up to the tunnel entrance. Vehicles enter the tunnel with a speed limit of either 60 km/h or 80 km/h [6]. For this study, the speed limit for HVs entering the tunnel is set at 70 km/h. In the experiment, vehicle arrivals follow a Poisson distribution with λ = 5, with a traffic volume of 720 pcu/h/l. The parameter values are presented in Table 2, and some IDM parameters, after calibration, align with those reported in the literature [44].

**Table 2:** Parameter values.

Parameter	Value	Parameter	Value
*V*_*m*_	33.34 m/s	*T*	1.5 s
*v*_*m*_	16.67 m/s	*v*_*d*_	19.45 m/s
*a*_*M*_	1.0 m/s^2^	s_0_	10.0 m
*a*_*d*_	2.0 m/s^2^	Δ*t*	1 s
*δ*	4		

Generally, two types of data can be utilized for assessing traffic safety: historical crash data and vehicle trajectory data [45]. While historical crash data provides valuable insights into crash frequency and severity, it has inherent limitations, as a site may lack crash data for certain time intervals yet still pose a risk. In contrast, vehicle trajectory data, typically extracted from traffic video, offers detailed microscopic information, including vehicle position, speed, and acceleration at second or sub-second intervals [46]. This granularity allows for the application of surrogate safety measures to assess potential conflict risks effectively.

Furthermore, existing studies predominantly employ Time to Collision (TTC) and Speed Standard Deviation (SD) as assessment indicators derived from vehicle trajectory data [47, 48]. In this paper, these metrics are employed to evaluate the safety improvements resulting from the speed optimization outcomes.

**Speed Standard Deviation (SD):** SD is used to describe the degree of dispersion or fluctuation within the vehicle speed dataset. Higher speed fluctuations often indicate more frequent acceleration and deceleration, which increases the risk of vehicle conflicts, such as sudden braking or rear-end collisions. Therefore, reducing speed fluctuations can decrease the likelihood of traffic accidents, thereby improving overall traffic safety. The SD of the traffic flow is calculated using Eq (34).

$$\sigma =\sqrt{\frac{\sum_{i=1}^{n}{\left(v_{i}-\bar{v}\right)}^{2}}{n-1}}$$ (34)

where *n* represents the total number of vehicles; $\bar{v}$ is the average speed of all vehicles.

**Time to Collision (TTC):** TTC is the estimated time required for a collision to occur based on the current positions and velocities of the vehicles. At a given time, if the speed of the front vehicle *i*−1 is less than that of vehicle *i* (i.e., *v*_*i*−1_(*t*)<*v*_*i*_(*t*)), there exists a potential risk of a rear-end collision. Hence, it is crucial for vehicles *i* and *i*−1 to maintain a safe headway distance. The calculation of the parameter *TTC*_*i*,*i*−1,*t*_ is determined by Eq (35).

$$if\;v_{i-1}(t)<v_{i}(t)\;then\;{TTC}_{i,i-1}(t)=\frac{s_{i-1}(t)-s_{i}(t)-l}{v_{i}(t)-v_{i-1}(t)},\forall i,i-1\in I_{l}$$ (35)

where *l* is the vehicle length, which is set as 5 m.

**Fig 4:**
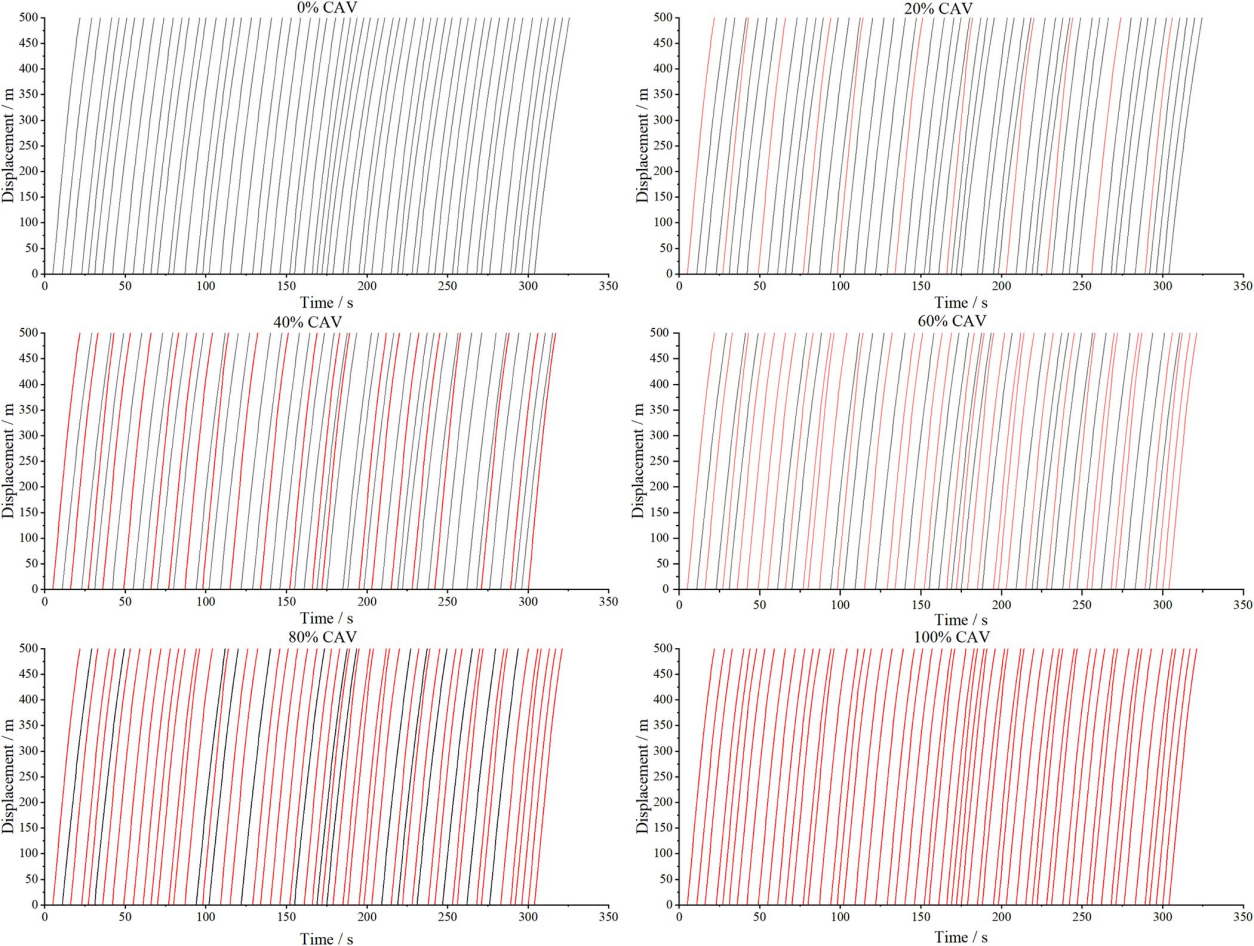
Vehicle trajectory under different penetration rates of CAVs.

**Fig 5:**
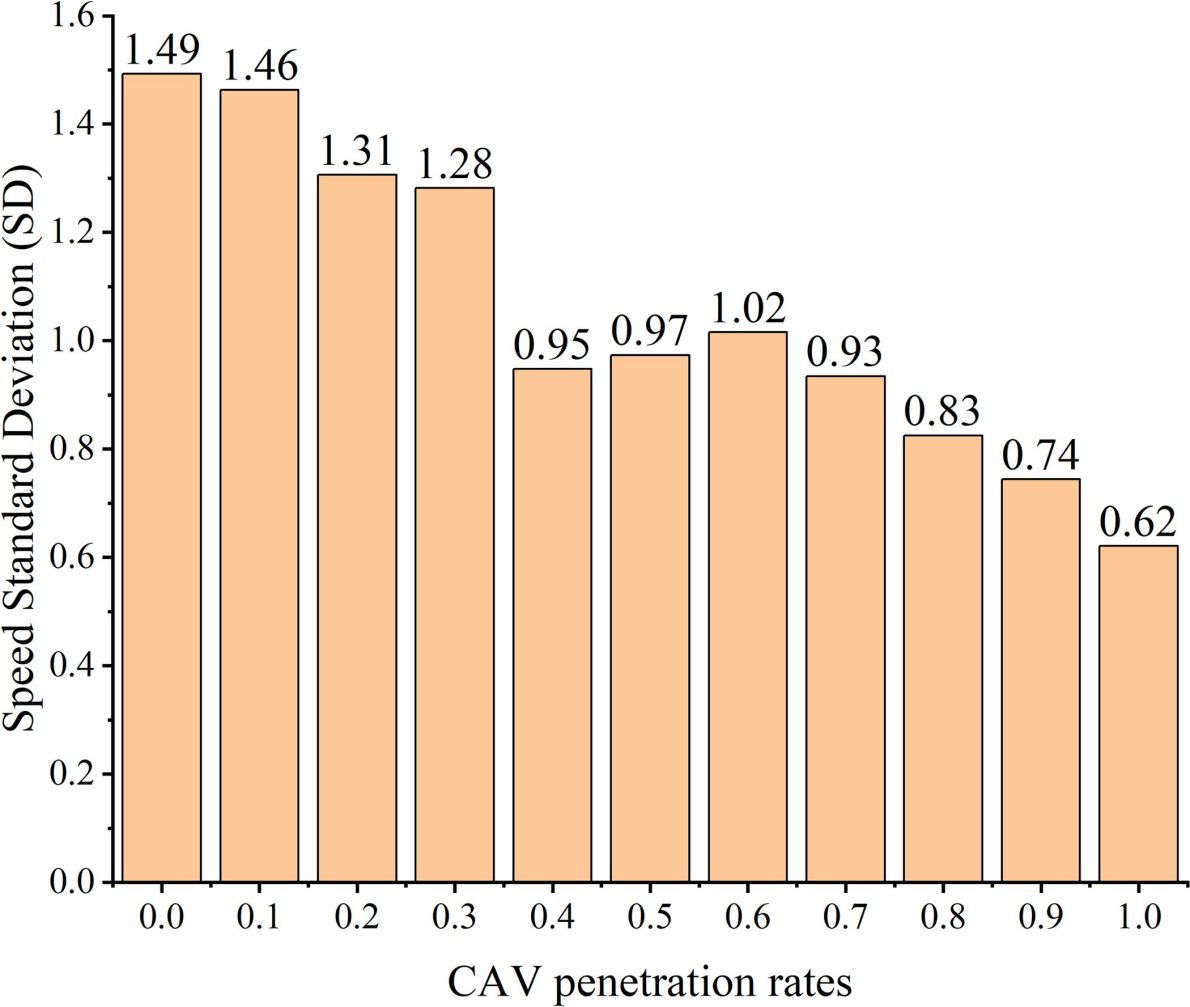
SD under different penetration rates.

**Fig 6:**
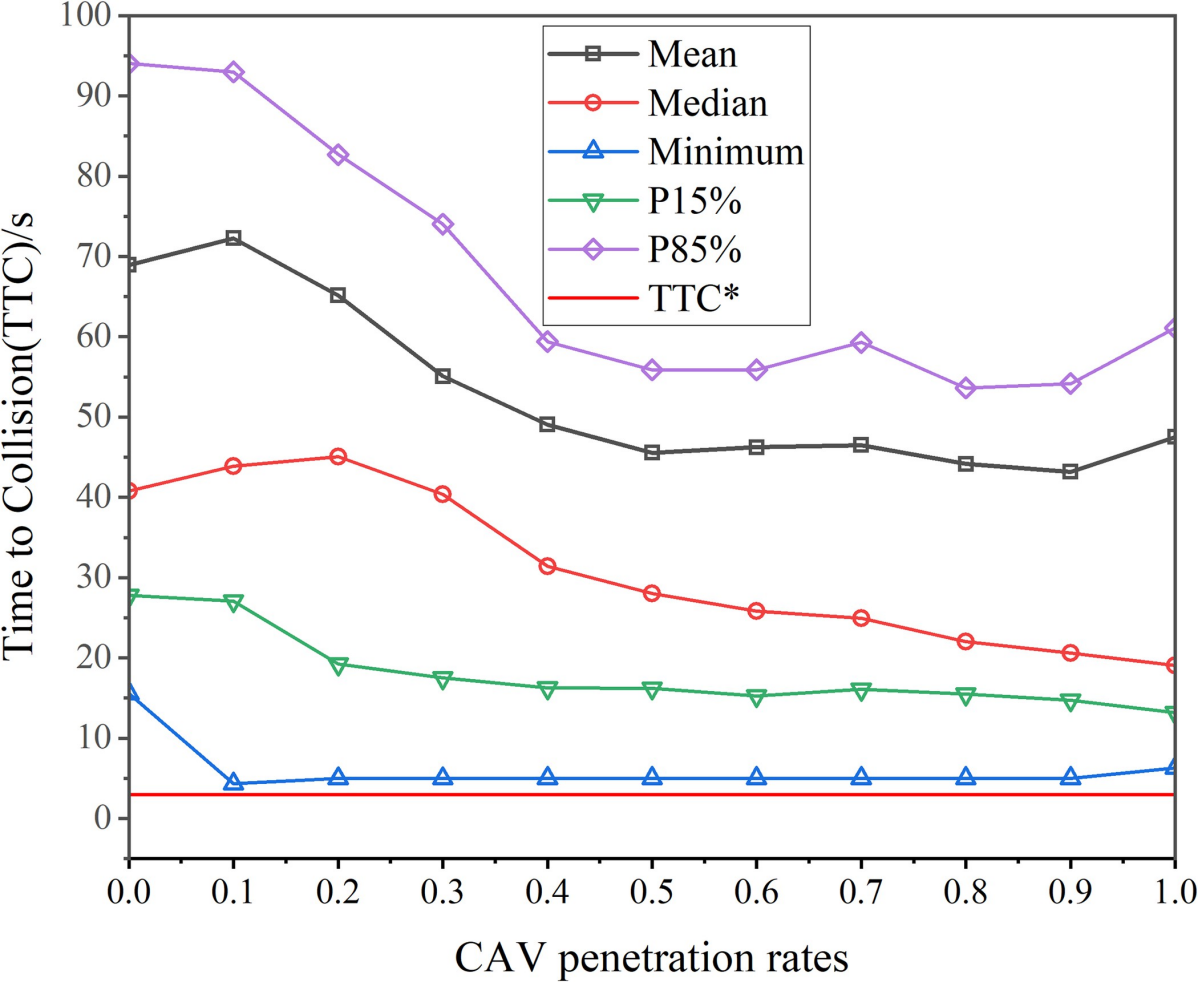
Statistical results of TTC under different penetration rates of CAVs.

The vehicle speed trajectories were computed for CAV penetration rates ranging from 0% to 100% in increments of 10%. The subplots in Fig 4 illustrate the space-time trajectories for CAV penetration rates of 0%, 20%, 40%, 60%, 80%, and 100%. In the figure, black lines represent the trajectories of HVs, while red lines denote the trajectories of CAVs. As observed in Fig 4, vehicle trajectories are smoother when the CAV penetration rate is either 0% or 100%, indicating improved traffic flow stability in a homogeneous traffic environment. The findings align with theoretical expectations. Safety indices, including SD and TTC, were calculated based on vehicle trajectories. The SD is depicted in Fig 5, while Mean TTC, Median TTC, and Minimum TTC are shown in Fig 6.

In this study, we calculated the Speed Standard Deviation (SD) for all vehicles after optimizing CAV speeds. As shown in Fig 5, increasing the CAV penetration rate results in a significant decline in the overall SD. A smaller SD indicates more consistent vehicle speeds in mixed traffic flow, which helps reduce the likelihood of traffic accidents. This suggests that the proposed speed control scheme has the potential to enhance traffic safety at the entrance zones of expressway tunnels. Notably, at a CAV penetration rate of 0%, the SD is 1.49. As the penetration rate increases to 100%, the SD decreases by 0.87, representing a substantial 58.38% reduction. Additionally, we computed the TTC per second for adjacent vehicles and presented statistical measures such as mean, median, minimum, etc., as depicted in Fig 6.

As shown in Fig 6, the minimum values of TTC at different CAV penetration rates consistently exceed the threshold value denoted as TTC*, which ranges from 1 to 3 seconds [49]. However, metrics such as the mean, median, and minimum TTC exhibit a decreasing trend. This trend can be explained by the fact that, while a larger TTC indicates a greater safe distance between vehicles, once the minimum TTC exceeds the specified threshold, the contribution of larger TTC values to safety becomes marginal. This marginal benefit may lead to a reduction in traffic efficiency.

**Fig 7:**
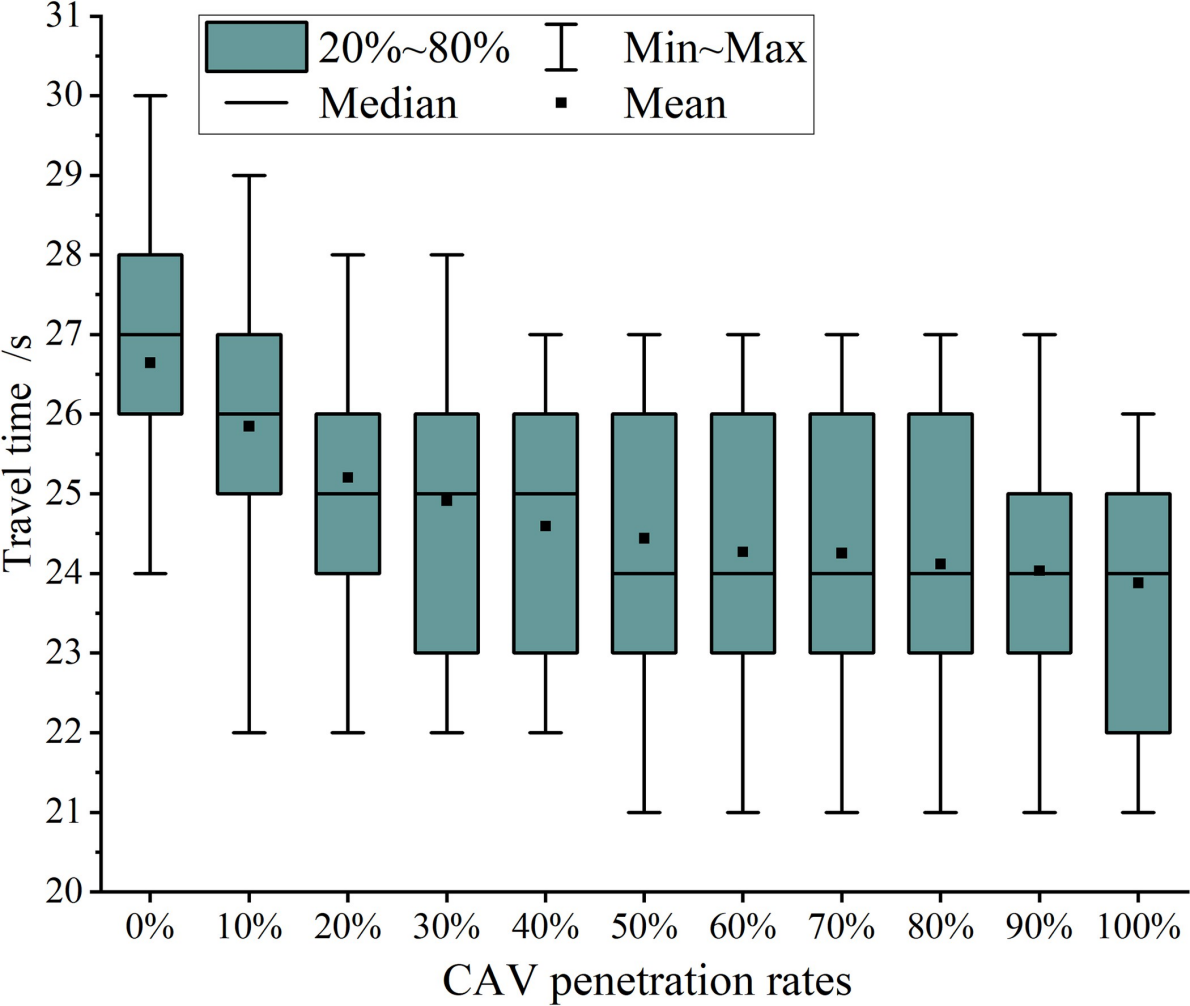
Vehicle travel time.

In this study, the objective function of the CAV optimization model consists of the weighted sum of speed fluctuations and the time taken to reach the tunnel entrance. Vehicles aim to reach the tunnel entrance as quickly as possible while maintaining a safe following distance behind the preceding vehicle. According to Eq (35), both the displacement and velocity differentials between adjacent vehicles decrease, leading to a reduction in TTC. It is important to note, however, that despite the overall reduction in TTC, the minimum values consistently remain above TTC*. Fig 7 illustrates the time taken by vehicles to traverse the speed control zone at different CAV penetration rates.

Two key conclusions can be drawn from Fig 7: Firstly, as the CAV penetration rate increases, both the average and maximum times for vehicles to reach the tunnel entrance decrease significantly. At a 0% CAV penetration rate, the average travel time is 26.64 seconds, and the maximum travel time is 30 seconds. In contrast, at a 100% CAV penetration rate, these times decrease to 23.88 seconds and 26 seconds, representing reductions of 10.37% and 13.34%, respectively. This reduction is attributed to the optimization goal of the CAV speed model, which aims to minimize the time required for vehicles to reach the tunnel entrance. As the CAV penetration rate increases, more vehicles benefit from this optimization, resulting in reduced travel times within the speed control zone.

Secondly, as the penetration rate increases from 0% to 40%, the mean travel time of vehicles decreases significantly, with an 8.27% reduction. However, as the penetration rate continues to increase, the rate of reduction slows. From a penetration rate of 50% to 100%, the reduction rate is only 2.29%. This suggests that beyond a 40% penetration rate, most HVs begin to adjust their speeds according to the trajectories of the surrounding CAVs.

The primary objective of this work is to enhance both safety and efficiency, with a particular emphasis on implementing appropriate speed control measures. In addition to discussing safety and efficiency, we argue that it is equally important to consider fuel consumption and pollutant emissions due to their strong connection with traffic management and vehicle speed regulation [50]. By conducting an in-depth analysis of fuel consumption and emissions, we underscore the broader benefits of speed control, including its positive environmental impact. These additional insights aim to provide readers with a more comprehensive understanding of the subject.

This paper uses the VT-micro model—a microscopic emission and fuel consumption estimation model—to calculate real-time fuel consumption and emissions of nitrogen oxides (NOx), carbon monoxide (CO), and hydrocarbons (HC) for vehicles. The VT-micro model is a polynomial regression model that relies on vehicle speed and acceleration as input parameters [51]. This model simplifies the computation process by eliminating the need for secondary calculations, thereby reducing potential errors. The expression for the VT-micro model is as follows:

$${MOE}_{e}=e^{\sum_{i=0}^{3}\sum_{j=0}^{3}\left(K_{i,j}^{e}∙u^{i}∙a^{i}\right)}e\in \{Fuel,NOX,HC,CO\}$$ (36)

Where *MOE*_*e*_ is instantaneous fuel consumption or emissions rate; $K_{i,j}^{e}$ is model of regression coefficient; *u* and *a* indicate instantaneous speed and instantaneous acceleration, respectively.

**Fig 8:**
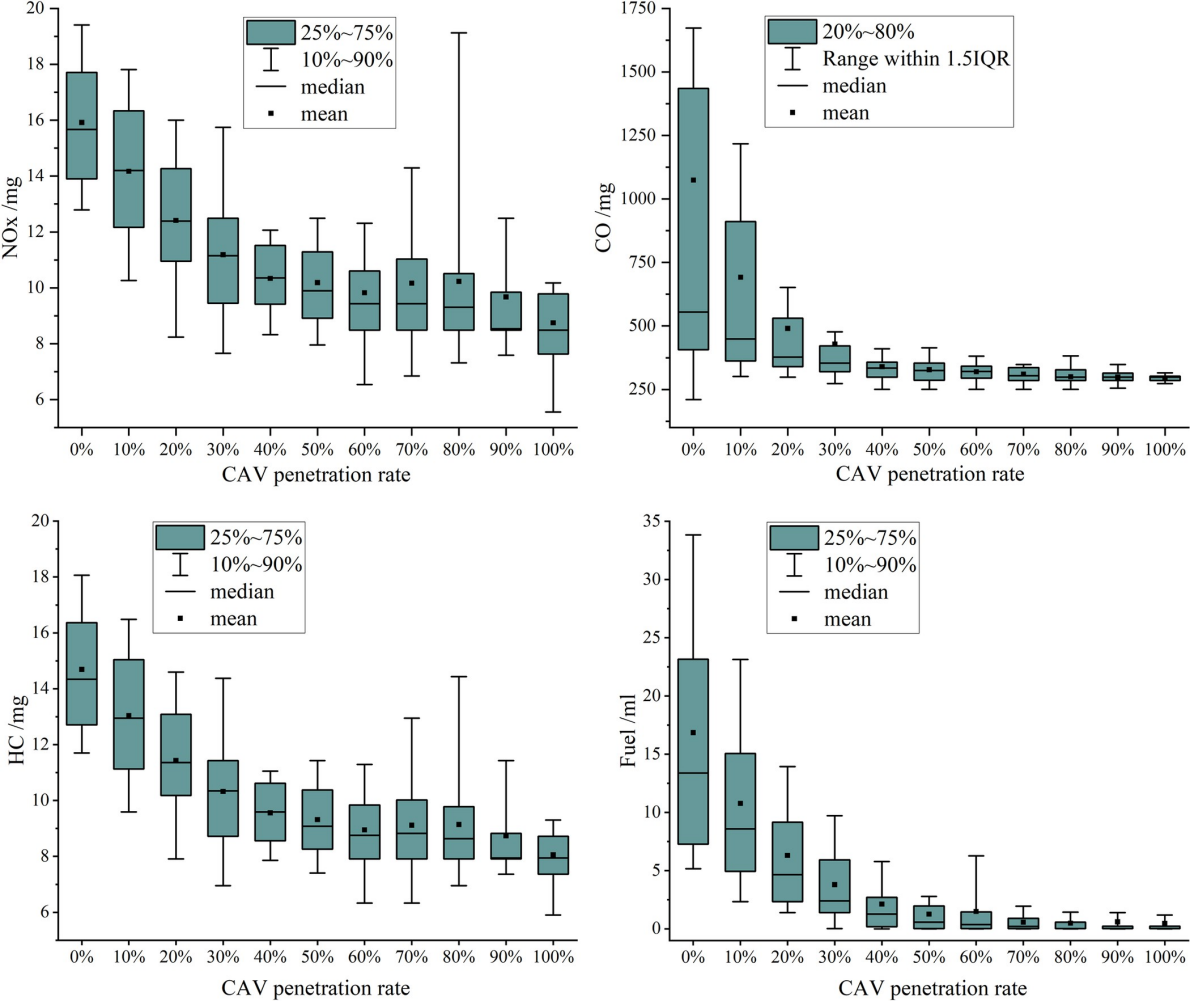
Vehicle fuel consumption and pollutant emissions at different penetration rate of CAVs.

Based on vehicle trajectories, fuel consumption and pollutant emissions for vehicles were calculated, with the results plotted in Fig 8. It is evident that as the CAV penetration rate increases, both fuel consumption and pollutant emissions decrease significantly. These findings align with theoretical expectations, as vehicles with shorter travel times and reduced speed fluctuations tend to exhibit lower fuel consumption and produce fewer pollutant emissions.

### 5.2 Sensitivity analysis

**Fig 9:**
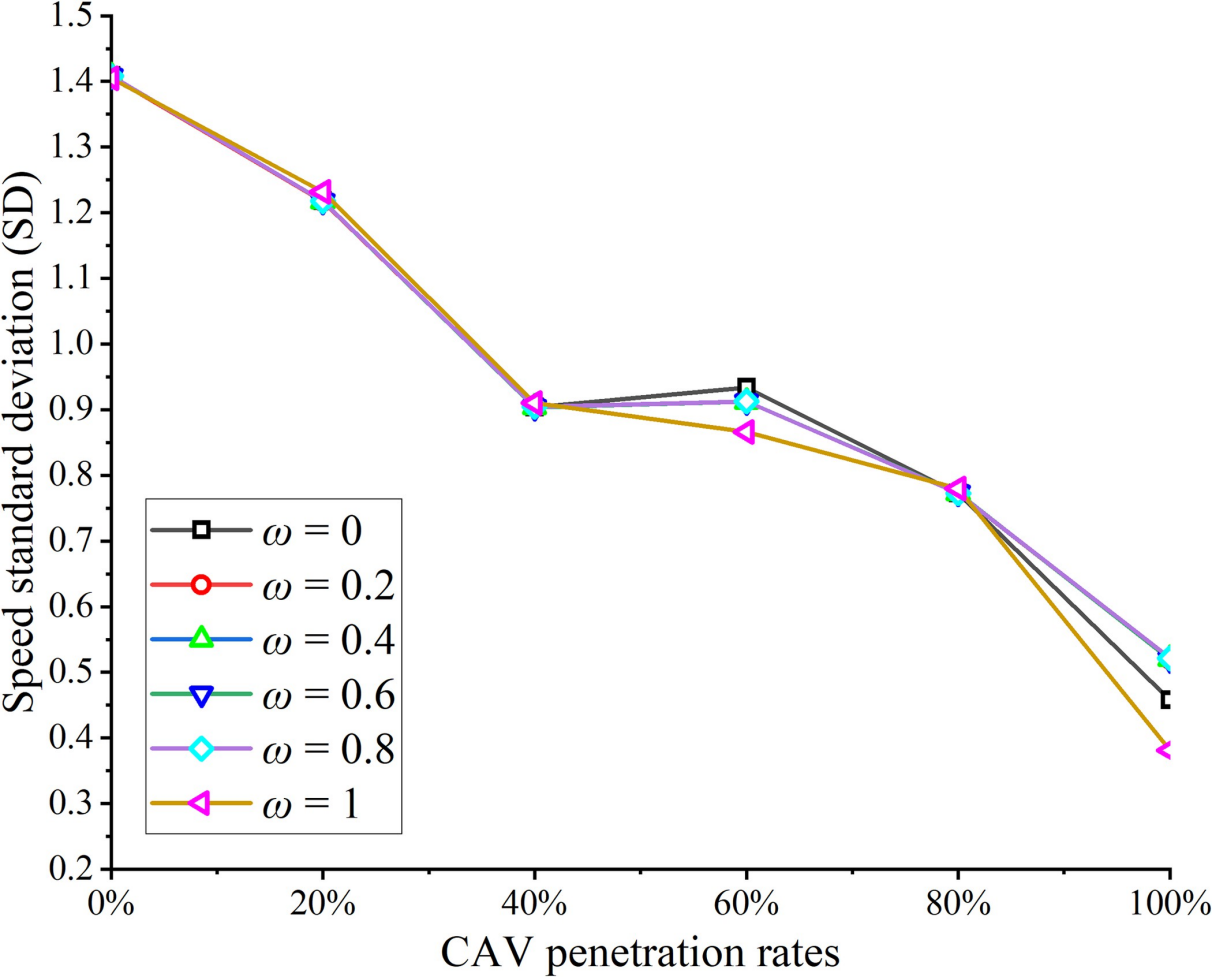
SD under different weights.

**Fig 10:**
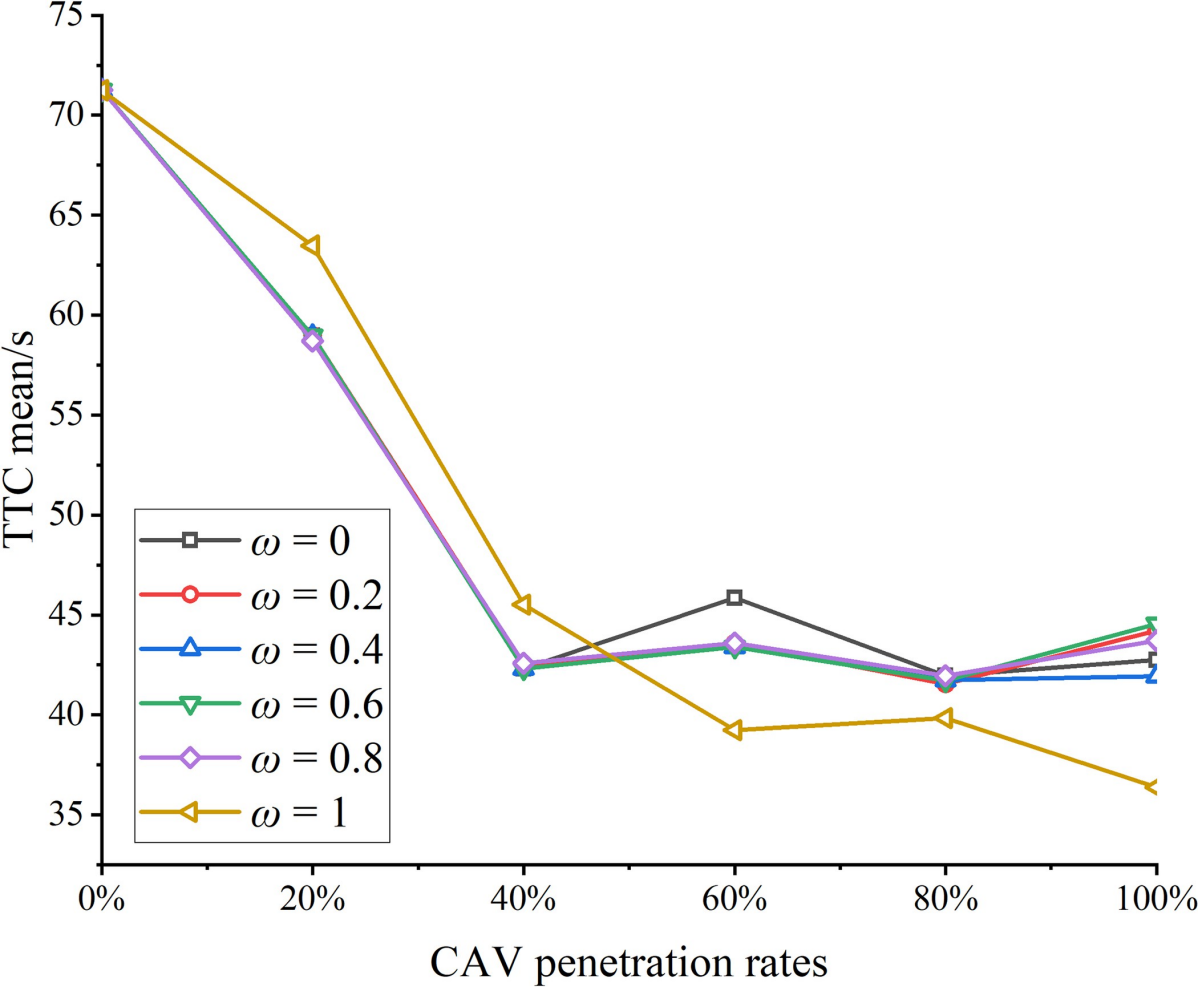
Mean TTC under different weights.

This section presents a sensitivity analysis of the weights in the objective function of the CAV speed optimization model. The weights (w) were varied from 0 to 1, and the SD and mean TTC were calculated for different weight configurations. The results are shown in Figs 9 and 10, leading to the following conclusions:

As the CAV penetration rate increases, both SD and mean TTC exhibit a significant decrease across various weight settings. This reduction is attributed to the growing number of CAVs optimizing their speeds, emphasizing the critical role of speed optimization in enhancing overall traffic safety.

The effect of penetration rate on SD and mean TTC is more pronounced when the CAV penetration rate is between 0% and 40%, consistent with the findings presented in Fig 7. Beyond 40%, most HVs adjust their speeds based on the trajectories of surrounding CAVs.

Variations in weights within the 0–0.4 range have minimal impact on both SD and mean TTC. This is because, at lower CAV penetration rates, the CAV speed optimization model is invoked less frequently. Consequently, the weights in the optimization model have a minor influence on traffic flow under these conditions.

At a 100% CAV penetration rate, the SD and mean TTC values for different weights are summarized in Table 3. An increase in weight generally leads to a decrease in both SD and mean TTC. For instance, when the weight increases from 0 to 1, SD and mean TTC decrease by 16.70% and 14.89%, respectively. However, this change does not follow a consistent pattern. This is due to the nature of the objective function in the CAV speed optimization model, where minimizing the speed difference with the preceding vehicle and minimizing the time to reach the tunnel entrance are not mutually exclusive. In some cases, increasing the vehicle speed reduces both the speed difference and the time required to reach the tunnel entrance.

**Table 3:** SD and mean TTC under different weights.

Safety evaluation index	ω = 0	ω = 0.2	ω = 0.4	ω = 0.6	ω = 0.8	ω = 1
SD	0.45777	0.51998	0.52071	0.51984	0.52159	0.38129
Mean TTC	42.76	44.21	41.94	44.56	43.72	36.39

## 6 Conclusion

The paper introduces a framework for a speed guidance model designed for expressway tunnel entrance zones in a mixed traffic environment. A CAV speed optimization model, with no more than five distinct stages, is developed to minimize speed fluctuations and the time taken to reach the tunnel entrance. The model includes constraints for safety, speed limits, and timing. Additionally, the IDM is used to calculate the speed trajectory of HVs. The speed optimization model is formulated as a MINLP, which is solved using the BONMIN solver through AMPL. Safety indicators, such as TTC and Speed SD, are selected to evaluate the model’s effectiveness in improving safety at tunnel entrances.

Case study results show a significant decrease in SD as the CAV penetration rate increases, with a 58.38% reduction from 0% to 100%. The impact on SD and mean TTC is most pronounced when the CAV penetration rate is between 0% and 40%, compared to rates above 40%. The minimum TTC values at different CAV penetration rates consistently exceed the safety threshold TTC*, confirming the effectiveness of the proposed control method in enhancing traffic safety. Sensitivity analysis further supports these findings. Additionally, the VT-micro model is used to calculate fuel consumption and emissions, demonstrating the proposed control method’s ability to reduce fuel use and pollution.

This study focuses on optimizing CAV speeds at expressway tunnel entrances under mixed traffic conditions to improve overall traffic safety. However, further research is needed. Firstly, this study focused on single-lane scenarios. Future research should examine multi-lane environments and include cooperative lane-changing models to better understand how lane-changing behaviors impact traffic safety and overall traffic dynamics. Studies such as Ma et al. (2022), which address speed guidance and lane-changing on highways, can serve as a useful reference [52]. Secondly, this study primarily focused on speed optimization without addressing communication latency issues. Future research, as referenced in [53], should investigate the effects of communication latency on speed optimization and propose strategies to mitigate these impacts. Finally, similar to most studies, this study did not account for the heterogeneous driving behaviors of human-driven vehicles. In future research, as suggested by references [54], HV driving behaviors could be categorized into three types—cautious, timid, and aggressive—based on driver styles. The IDM parameters can then be calibrated to reflect these varying driver behaviors.
